# Comparative Transcriptomics between *Synechococcus* PCC 7942 and *Synechocystis* PCC 6803 Provide Insights into Mechanisms of Stress Acclimation

**DOI:** 10.1371/journal.pone.0109738

**Published:** 2014-10-23

**Authors:** Konstantinos Billis, Maria Billini, H. James Tripp, Nikos C. Kyrpides, Konstantinos Mavromatis

**Affiliations:** 1 Microbial Genome and Metagenome Program, DOE-Joint Genome Institute, Walnut Creek, California, United States of America; 2 Group of Prokaryotic Cell Biology, Max Planck Institute for Terrestrial Microbiology, Marburg, Germany; 3 Computational Biology Group, Celgene Corp, San Francisco, California, United States of America; 4 European Molecular Biology Laboratory, European Bioinformatics Institute (EMBL-EBI), Wellcome Trust Genome Campus, Hinxton, Cambridge, United Kingdom; 5 Department of Genetics, Development and Molecular Biology, Aristotle University of Thessaloniki, Thessaloniki, Greece; 6 Department of Biological Sciences, Faculty of Science, King Abdulaziz University, Jeddah, Saudi Arabia; 7 Faculty of Biology, Philipps-Universität, Marburg, Germany; University of Freiburg, Germany

## Abstract

*Synechococcus* sp. PCC 7942 and *Synechocystis* sp. PCC 6803 are model cyanobacteria from which the metabolism and adaptive responses of other cyanobacteria are inferred. Using stranded and 5′ enriched libraries, we measured the gene expression response of cells transferred from reference conditions to stress conditions of decreased inorganic carbon, increased salinity, increased pH, and decreased illumination at 1-h and 24-h after transfer. We found that the specific responses of the two strains were by no means identical. Transcriptome profiles allowed us to improve the structural annotation of the genome i.e. identify possible missed genes (including anti-sense), alter gene coordinates and determine transcriptional units (operons). Finally, we predicted associations between proteins of unknown function and biochemical pathways by revealing proteins of known functions that are co-regulated with the unknowns. Future studies of these model organisms will benefit from the cataloging of their responses to environmentally relevant stresses, and improvements in their genome annotations found here.

## Introduction

Cyanobacteria are considered to be among the oldest organisms evolutionarily, given that putative microfossils attributed to cyanobacteria are 3.5 billion years old [1]. *Synechocystis* sp. strain 6803 (referred hereafter as *Synechocystis*) and *Synechococcus* sp. PCC 7942 (referred hereafter as *Synechococcus*) are two of the most well-studied cyanobacterial model organisms. Like other cyanobacteria, these organisms are capable of oxygenic photosynthesis. Most recently, they have attracted a great deal of attention because of their potential for photobiological production of biofuels and carbon sequestration [2], [3]. Because of their great importance, the complete sequence of their genomes were among the first photosynthetic bacteria to be elucidated [4], [5]. Despite their similarities, both strains exhibit a number of differences in their response to environmental stresses. For example, even though *Synechocystis* and *Synechococcus* are both freshwater unicellular strains, *Synechocystis* can tolerate elevated concentrations of NaCl better than *Synechococcus*. Furthermore, *Synechococcus* is an obligate photoautotroph [6], while *Synechocystis* is a facultative photoautotroph [7].

Data obtained with RNA-seq have been shown to have higher dynamic range than microarray chips and are useful for improving genome structural annotations [8], [9], [10]. In this study we used RNA-seq to characterize the transcriptomes of *Synechocystis* sp. PCC 6803 and *Synechococcus* sp. PCC 7942 under various stress conditions in order to reveal their transcriptional responses to environmental stress and to improve their genome annotations. The specific stresses we studied were increased salinity, increased pH, mild carbon limitation, decreased temperature, and decreased light. Although increased pH is considered a “stress”, prior research has shown a coincidence of high pH with cyanobacterial blooms, as they draw down CO_2_ during photosynthesis and shift the aquatic bicarbonate buffering system toward consumption of H^+^ [11]. We also had a broader interest in the relative uses of respiration and photosynthesis for energy acquisition across all types of stress, given that cyanobacteria can acquire energy from either source.

Our main goals in this study were to: a) improve the structural annotation of the two genomes by correcting already annotated genes and identifying missed genes (including anti-sense) and transcriptional units (operons); b) improve the functional annotation of genes and pathways using gene co-expression information; and c) identify alterations in the short and long term expression patterns of genes in energy production pathways under various stress conditions.

The complete sequence and annotation of both strains is available through the Integrated Microbial Genomes (IMG) [12], [13]. The expression data are also made available through IMG, and together with the transcriptome analysis presented in this paper, they serve as a valuable resource for further studies of the transcriptional mechanisms of these organisms.

## Materials and Methods

### Cyanobacterial growth and conditions

*Synechococcus* and *Synechocystis* were grown and sampled under an initial condition (reference), then subjected to five stress conditions, which were sampled at 1-hour and 24-hours (Table 1). To establish the reference condition, cells were grown in standard BG-11 medium in sterile glass flasks on a table top orbital shaker with cotton caps to allow for proper CO_2_ incorporation. Except where noted, all cultures were grown at 30°C, 100 rpm, light 100 µE×m-2×sec^−1^, and 3% CO_2_. To establish the reference condition a 50 ml BG-11 flask was inoculated from a stock cyanobacterial culture and grown to late exponential state (D_730_∼8). Cells from this culture were then grown in a new 100 ml BG-11 flask containing media adjusted to pH 7.5 using 20 mM HEPES-KOH up to OD_730_ = 2.0 (early to mid exponential phase), at which time the reference condition was defined and sampled. The following stresses were then applied as described below.

**Table 1:** Growth Conditions applied on the two organisms and their run ids from NCBI SRA.

	*Synechococcus sp. PCC 7942*		*Synechocystis sp. PCC 6803*	
Reference	S7-Ref (*SRR071374*)		S6-Ref (*SRR071349*)	
	1-hour post stress	24-hours post stress	1-hour post stress	24-hours post stress
Increased salinity (S)	S7-S 1-h (*SRR071375*)	S7-S 24-h (*SRR071379*)	S6-S 1-h (*SRR071348*)	S6-S 24-h (*SRR071356*)
Reduced light (L)	S7-L 1-h (*SRR071378*)	S7-L 24-h (*SRR071384*)	S6-L 1-h (*SRR071360*)	S6-L 24-h (*SRR071355*)
Lower temperature (T)	S7-T 1-h (*SRR071385*)	S7-T 24-h (*SRR071382*)	S6-T 1-h (*SRR071358*)	S6-T 24-h (*SRR071352*)
Increased pH (P)	S7-P 1-h (*SRR071377*)	S7-P 24-h (*SRR071383*)	S6-P 1-h (*SRR071357*)	S6-P 24-h (*SRR071350*)
Carbon Dioxide deficiency (C)	S7-C 1-h (*SRR071380*)	S7-C 24-h (*SRR071372*)	S6-C 1-h (*SRR071353*)	S6-C 24-h (*SRR071359*)

- Increased NaCl concentration: 200 mM for *Synechococcus* and 550 mM for *Synechocystis*. Based on different threshold point of salt tolerance between the two strains determined by previous studies [13], [14], [15], [16], [17], [18].
- Increased pH: to 10.0 from 7.5. Once the reference condition was established, we pelleted the cells, washed them twice with BG-11+20 mM Bis-Tris-Propane- MES pH 10.0 and resuspended them in 100 ml BG-11+20 mM Bis-Tris-Propane- MES pH 10.0.
- Reduced light: 20 µE×m-2×sec^−1^ from 100 µE×m-2×sec^−1^.
- Decreased temperature: to 20°C from 30°C.
- Mild Carbon limitation: At OD_730_ = 2.0 we closed the CO_2_ circulation, left the chamber open for 5 min to allow for removal of residual CO_2_, then allowed the culture to continue growth.

25 ml samples were collected 1-h and 24-h after the application of each stress condition.

### RNA isolation - Sequencing

Ribosomal RNA was depleted from total RNA with MicrobExpress (Ambion/Life technologies). Purified mRNA was then fragmented using RNA fragmentation reagents (Ambion). Fragmented RNA served as the template for 1st strand cDNA synthesis. Reverse transcription was performed using SuperScript II Reverse Transcription (Invitrogen), with random hexamer as primer. This was followed by second strand synthesis using dNTP mix where dTTP is replaced by dUTP. Double stranded cDNA fragments were then blunt-ended, A tailed and ligated with Illumina adaptors. Digestion of dUTP was then performed using AmpErase UNG (Applied Biosystems).

Single Strand RNA adapter (5′-ACACUCUUUCCCUACACGACGCUCUUCCGAUCU-3′) was unidirectionally ligated to the 5′ end of the mRNA using T4 RNA Ligase I (New England BioLabs). The first strand of cDNA was synthesized using a fusion primer (5′-GTGACTGGAGTTCAGACGTGTGCTCTTCCGATCTNN–NNN*N-3′) and SuperScript II reverse transcriptase (Life technologies). After cleanup with Agencourt AMpure SPRI beads, 18 cycles of PCR reaction was carried out with KAPA HiFi DNA Polymerase and the Illumina TruSeq PCR primers. A gel size selection procedure was used to collect a library of products ranging from 200 to 700 bp in size. Sequencing data was generated from an Illumina GAII Sequencer. 5′ enriched libraries were constructed according to [20] and sequence data was generated from an Illumina MiSeq benchtop sequencer.

### Data quality and mapping

The quality of the sequenced reads was first assessed using the Bioconductor shortRead package [21]. The majority of the reads (>95%) were of high quality with the median Phred quality above 38 in all samples. Reads were aligned to the reference genome sequence or the corresponding strain provided by IMG [12] (Table S1). The Burrows-Wheeler Alignment tool (BWA) [22] was used for the mapping, allowing default parameters and mapping to multiple locations. Artemis [23] and Gbrowse [24] were used to visualize the mapping results.

After the rRNA depletion step 66% of *Synechocystis* and 71% of *Synechococcus* reads mapped to ribosomal RNA genes and were not considered in downstream analysis (Table S1).

### Gene expression analysis

Transcript abundance was normalized using RPKM (Read Per Kilobase of transcript per million Mapped reads) (Table S2) [25]. In order to determine whether biases in the expression profile of each gene along its length were consistent across all conditions we calculated the Spearman correlation between each pair of conditions for each gene. For the majority (>90%) of the genes the correlation was consistently above 0.5, indicating either absence of sequencing biases or consistency across all conditions. The remaining 10% of the genes were poorly expressed (RPKM∼2.5*10^−9^). Gene set enrichment analysis was performed using the COG annotation provided by IMG. Statistical evaluation was performed using the Fisher's exact test with adjustment of the p-values for multiple comparisons using the Benjamini & Hochberg correction with FDR = 0.05.

Hierarchical clustering was performed using R hclust using Pearson correlation as distance metric and complete as the agglomeration method.

Differential gene expression analysis was performed using the R package edgeR [26]. P-values were adjusted with the Benjamini & Hochberg correction with FDR = 0.05. After selecting genes with an adjusted p-value<0.01 we added genes belonging to the same pathway if they exhibited similar logR_2_ values or belonged to the same TU (as defined previously).

### Annotation Improvement Techniques

Transcripts that mapped to intergenic regions were considered to confirm an unannotated gene if their coverage, defined as the number of reads mapped to any given nucleotide of this gene, was consistently 5 or more in at least 3 conditions, and the overall size of the transcript was greater than 200 bp. The latter threshold was selected to account for the exclusion of shorter transcripts during sequencing library preparation.

One five prime enriched library was used to identify putative starts of the transcripts on *Synechococcus*. This library was prepared from pooling RNA from all available samples of this strain. In order to identify the accuracy of the 5′ signals we first analyzed their intensity on a set of high quality predicted CDSs. This set was comprised of genes that had been assigned to COGs and that had at least 150 bp downstream of their 3′ end in order to avoid interference from trailing coverage of transcripts or operon structures. This allowed us to correlate the intensity of the 5′ peak to the coverage of the CDS. In order to avoid 5′ RNA-seq bias we also excluded the first 150 bps immediately after the 5′ peak in the computation of the correlation. A threshold correlation of 0.6 or better using the Passing & Bablok regression was used to find all 5′ peaks with similar correlation to their downstream transcript coverage. All the 5′ signals that passed the previous filter were assigned to a CDS at most 200 bps downstream of the signal.

Transcriptional Units (TU) or operons were defined as collections of tandem CDSs with intergenic distance less than 150 bps, common expression profiles under different conditions (i.e. Pearson correlation between all conditions >0.6) and, in the case of *Synechococcus*, absence of 5′ peaks assigned to genes inside the TU.

## Results and Discussion

### Whole genome expression

The total percentage of the two genomes, including intergenic and gene coding regions, covered by more than two RNA-seq reads from any condition was 98.6% for *Synechococcus* and 98.7% for *Synechocystis*. This can be attributed to (a) the high gene density (coding sequences cover 89.2% and 86.7% of the entire genome of *Synechococcus* and *Synechocystis* respectively). We observed that 99% of the coding bases were found to be covered by at least two reads, (b) expression of 5′ or 3′ UTRs or intergenic regions between genes belonging to operons, (c) the presence of transcripts in intergenic regions that have not been annotated. In any case, caution should be taken in interpreting these results, since they may also represent sequencing artifacts [27]. As described below, we tested these assumptions by examining the structural annotation of existing genes, specifically their 5′ start sites. We also examined the validity of novel transcripts.

### Structural annotation of existing genes

**Figure 1:**
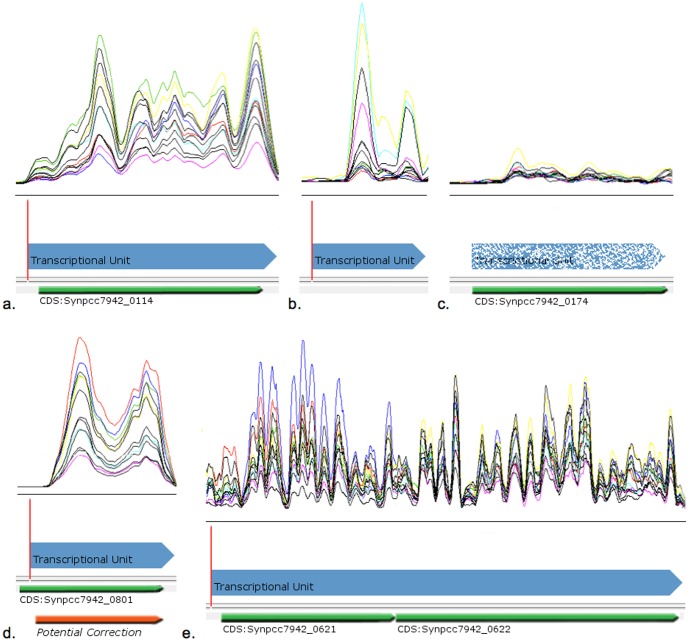
Regions of the genome combined with annotation, RNA-seq expression and 5′ peaks information. The colored lines represent the expression coverage of this region in each condition. Green arrows represent the existing annotation. 5′ peaks are indicated with a vertical red line, blue arrows that follow 5′ peaks denote the potential Transcriptional unit and follows the expression of this region. a. Example of a CDS of *Synechococcus* (Synpcc7942_0114) with one clear 5′ enriched indication, RNA-seq coverage and proper annotation. b. Example of a TU inside non-coding region with one clear 5′ peak. c. Example of an annotated CDS (Synpcc7942_0174) without 5′ peak and ambiguous RNA-seq coverage. d. Example of a CDS (Synpcc7942_0801) with evidence of structural misannotation since the 5′ peak is located 60 bps after the annotated start site. e. Two annotated genes with contiguous expression and a peak at the 5′ of the first gene suggesting that both CDSs are expressed together as one transcript forming an operon.

The 5′ enriched libraries of *Synechococcus* revealed that 792 of 2612 CDSs did not have a reliable 5′ signal. Of these 792, 522 were part of a predicted transcriptional unit (TU) and 270 were not. Those 270 CDSs (Figure 1c) were poorly expressed under all experimental conditions (Table S3). Interestingly, these genes have similarities to other genes in other genomes and many of them have hits to protein families (pfam, COG) and functional annotations. The 5′ enriched libraries also revealed 171 CDSs whose 5′ peak was downstream of the annotated start codon (Figure 1d) (Table 2).

**Table 2:** Summary of structural annotation for *Synechococcus* sp. PCC7942.

*Synechococcus sp. PCC 7942*	Number	Percent
Total Genes	2612	100%
Genes without conflicts	2441	93.45%
Genes with altered Start site	171	6.5%
Novel Genes	256	+9%
Total number of transcriptional units (TUs)	2090	100%
TUs with 5′ peaks	1820	87%
TUs without 5′ peaks	270	13%

Overall, a total of 375 TUs were identified in *Synechococcus* with two or more predicted CDSs encompassing a total of 897 CDS (Figure 1e). Similarly, *Synechocystis* is predicted to contain 311 TUs comprised of 754 genes.

### Novel transcripts

Using the coverage profiles, we looked for novel transcripts (i.e. transcripts not annotated in the current publicly available genome annotation) according to the method described in the materials and methods section. Due to size limitations imposed by the library preparation method, we restricted our prediction to transcripts of 200 bp or longer. We were able to predict 256 new transcripts in the *Synechocystis* and 127 in *Synechococcus* located in the intergenic regions – based on the current annotation. Approximately half of these transcripts returned significant hits (blast evalue <10^−5^) when compared to the nr protein database (File S1), suggesting that these genes were missed in the current annotation of these organisms, while the other half most likely represents novel transcripts of unknown product.

Additional novel transcripts were detected on the antisense strands, 930 in Synechocystis and 573 in *Synechococcus*. In the case of *Synechococcus* we were also able to further support the detection of antisense transcripts by identifying 5′ peaks supported by at least 5 reads, located in the antisense strand. Previous studies have detected expressed transcripts in the opposite (antisense) strand of protein coding genes in Bacteria and describe their role as a posttranscriptional mechanism to adjust mRNA levels [19], [20], [21]. However since our study was not designed to detect small size transcripts, we refrained from further exploring this prediction.

### Comparison of new features to previous studies

Our results were compared to other studies that report new features of cyanobacterial genomes [2], [31]. The predictions that are demonstrated in this study are similar to those reported in [31] but are not widely comparable against [2].

Compared to [31] TU predictions for *Synechococcus* completely agree in 183 cases and exhibit difference of at least one gene in 142 of our predictions. 243 TUs were not found in our study and we predicted an additional 50. TUs that were not predicted in our study typically either have low expression of intergenic regions between genes, or significant 5′ peaks were detected upstream of the CDSs, or the genes show different expression profiles in the studied conditions. The ncRNA genes predicted in [31] were compared against our combined novel transcript predictions resulting in identifying 301 common genes (out of the 700 of our predictions) with at least a 40% overlap in their sequence. Similar comparisons were accomplished between our and [2] predictions for *Synechocystis*, with an overlap of 324 out of 1185.

Comparison to [2] was limited to predicted transcripts with length greater than 200 bp, a limitation presented by the library preparation method used in our study. In that case the 46 out of 82 long asRNA and ncRNA in [2] are also present in our dataset.

Transcripts predicted by these studies but not in ours were manually inspected to identify the cause of the discrepancy. In all cases inspected, the reason for this discrepancy is due to either the lack of significant 5′ peak or expression levels below the thresholds set. These differences can be attributed to the different sequencing technologies and protocols that were applied and the amount of data collected. Further investigation of these candidate genes can help verify their role and understand the biology of the organisms.

### Gene expression and stress condition clustering

**Figure 2:**
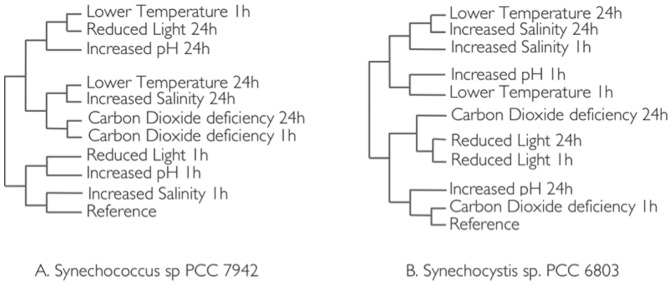
Hierarchical clustering of stress conditions for *Synechococcus* sp. PCC 7942 and *Synechocystis* sp. PCC 6803.

Unsupervised hierarchical clustering reveals the similarities and differences in stress response for the two study organisms (Figure 2). The strongest similarities with respect to time were carbon depletion at 1-h and 24-h in *Synechococcus* (Figure 2A) and reduced light at 1-h and 24-h in *Synechocystis* (Figure 2B). These time similarities indicate that the respective responses for the two organisms manifest themselves within 1-h and do not change significantly for the next 24-h. In addition, the greater distance between the reference condition and carbon stress cluster in *Synechococcus* and the distance between the reference condition and reduced light cluster in *Synechocystis* suggests that the immediate carbon stress response in *Synechococcus* is stronger than the immediate reduced light response in *Synechocystis*. The same kind of analysis leads to the conclusion that the pH response does in *Synechocystis* occurs largely within an hour. Clearly, different stresses elicit similar time responses in the gene expression patterns of the two organisms.

Unsupervised clustering can be used to reveal gene relationships based on the assumption that genes with similar expression profiles are functionally linked [32]. Based on this approach we were able to identify 6 proteins of unknown function (annotated as hypothetical proteins) in the current annotation, in *Synechocystis* and 1 in *Synechococcus*, which exhibited high correlation (Pearson correlation >0.9) to proteins of known function (Table S4). The six proteins in *Synechocystis* are smaller proteins (<145 amino acids) related to energy and nitrate metabolism. In one case (ssl5045), transcriptomics confirmed the existence of the small gene corresponding to a protein of 60 amino acids, which is located in the intergenic space in the July 2012 draft update of the genome. The *Synechococcus* protein was co-regulated with annotated twitching motility proteins. It needs to be noted that these associations do not reveal the exact function of these proteins, but rather indicate a common regulation and/or a functional correlation among them, and require additional experiments to verify or reject these hypotheses.

### Differential gene expression

Pairwise comparisons between the reference and stress conditions, as well as between the 1-h and 24-h conditions revealed a relatively small number of genes that exhibit either increased or decreased transcription levels (an arbitrary threshold of logR_2_ greater than 2 was selected). The genes and their classification to COG functional groups are shown in detail in 2.

In order to further associate the number of up/down-regulated genes to known pathways we performed gene set enrichment analysis using the COG functional classification. This analysis also revealed a different pattern of response between the two organisms.

Some responses to alkaline pH stress that we observed in both *Synechococcus* and *Synechocystis* are consistent with previous studies [11]. Alkaline pH in freshwater systems is thought to be caused by high levels of photosynthesis, which drive down dissolved CO_2_ levels. Lowered CO_2_ shifts the bicarbonate buffering system in the direction of consuming hydrogen, resulting in raised pH, as the hydrogen combines with bicarbonate to form CO_2_ and water, thus maintaining equilibrium. Cyanobacteria, specifically *Synechocystis*, have been shown to acclimate in alkaline pH by spending energy to acquire and concentrate scarce CO_2_ and bicarbonate, allowing them to grow and outcompete other photosynthesizers at high pH. Consistent with these studies, we found that *Synechococcus* gene sets related to cell wall biosynthesis and cell motility/signal transduction were enriched with upregulated genes under alkaline pH stress at 1-h and that gene sets for energy production and translation were upregulated at 24-h. We also found upregulation of ribosomal genes and translation in *Synechocystis* after 1-h. Certainly energy production, motility and translation are indicators of high metabolic activity, such as occurs during the exponential growth phase of blooms. We speculate that cell wall biosynthesis reflects either increased growth or physical restructuring of cell walls to tolerate alkaline conditions [11].

Other results of gene set enrichment analysis are itemized below.

- In *Synechococcus* elevated NaCl after 1-h causes upregulation of genes related to signal transduction and downregulation of nutrient transport (particularly nitrate and phosphate). After 24-h translation is upregulated, particularly the operon Synpcc7942_2203 - Synpcc7942_2235, and genes related to transcription, replication and signal transduction are downregulated. In *Synechocystis*, elevated NaCl after 1-h causes upregulation of translation and replication, downregulation of genes classified in cell wall biogenesis, and downregulation of translation after 24-h. These results, however, are not directly comparable since the two organisms exhibit different levels of tolerance for NaCl.
- In *Synechococcus* CO_2_ depletion after 1-h results in higher expression of energy production genes and intracellular trafficking genes, and lower expression of genes related to signal transduction. After 24-h energy conversion related genes remain enriched. In *Synechocystis* CO_2_ depletion after 24-h results in lower expression of genes involved in energy production, basic metabolism and inorganic transport.
- In *Synechococcus* lower temperature after 1-h increases expression levels of genes related to chaperones and heat shock proteins, and decreases expression of signal transduction related genes. After 24-h the transcription levels of energy production and translation related genes are elevated while transcription and signal transduction are lowered. In *Synechocystis* lower temperature after 1-h upregulates genes related to replication, which remain elevated after 24-h. Translation appears to be elevated after 24-h as well. Interestingly transposases are also elevated both after 1-h and 24-h.
- In *Synechococcus* lowered light after 1-h caused increased expression levels of genes related to energy production, and decreases the expression levels of genes related to nutrient transport (phosphate, nitrate, sulphate). After 24-h, lowered light increases expression of genes associated with post-translational modifications, and decreases signal transduction mechanisms. In *Synechocystis* lowered light after 1-h and 24-h decreases expression of genes for metabolic pathways and protein translation.

Overall, many stress conditions affected expression of protein translation genes. Expression of genes associated with protein translation has been shown to vary under stress conditions [33] although not to the degree reported here. Increased expression of ribosomal proteins and ATP synthase, has been associated with favorable growth conditions, including light-versus-dark transition and log-phase growth versus stationary-phase growth [11]. This could be a reason for their increased expression in several of the 24-h conditions, particularly in the case of *Synechococcus*. In the case of salt stress it has been suggested that high ionic strength might destabilize ribosomes, requiring replacement of ribosomal proteins to maintain ribosomal activity [34].

### Photosynthetic and respiratory energy generation

**Figure 3:**
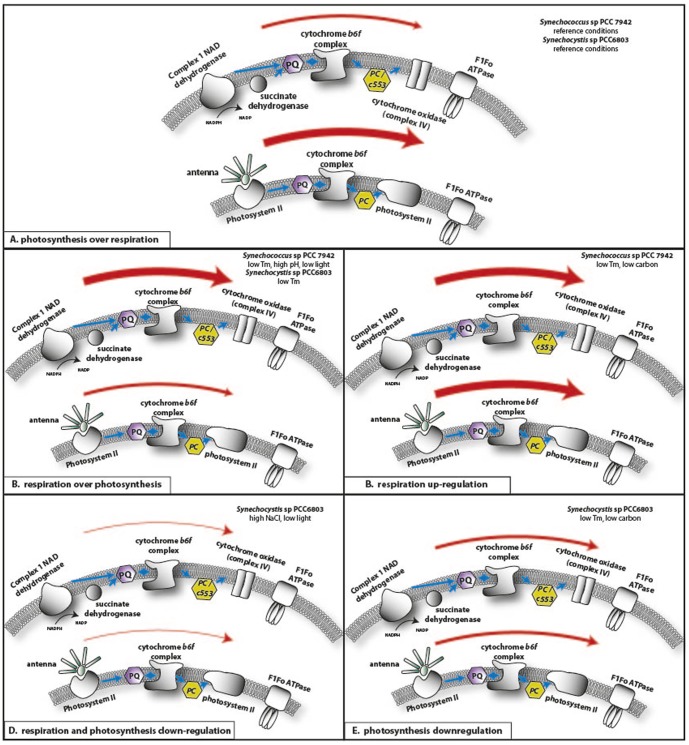
Model: How the relative expression of components of the electron transport chains of photosynthesis and respiration may affect the energy flow at specified time points of stress acclimation. The thickness of the respective arrows represents the suggested intensity of the energy flow. The conditions that apply to the panel are shown in the upper right of the panel. Panel A. Reference conditions, in the presence of light the photosynthetic electron transport chain has a higher capacity of electron flow than the respiratory chain. Panel B. Conditions where photosynthetic genes are upregulated and respiratory ones are downregulated (*Synechoccus*: alkaline pH (1-h & 24-h), low light (1-h & 24-h), low temperature (24-h), and *Synechocystis*: low temperature (24-h)). Panel C. Conditions where respiratory genes are upregulated (*Synechococcu*s: low temperature (24-h) and carbon limitation (1-h)). Panel D. Conditions where photosynthetic and respiratory genes are both downregulated (*Synechocystis*: low light (1-h & 24-h), elevated salinity (1-h)). Panel E. Conditions where photosynthetic genes are downregulated (*Synechocystis*: low temperature (1-h), and carbon limitation (24-h)). For simplicity, the two electron transport chains are represented in two different membrane sections, even though respiration is met in the cytoplasmic and both respiration and photosynthesis are intertwined in the thylakoid membrane. The intersection of the two pathways in the thylakoids is more apparent in *Synechocystis* than *Synechococcus*, where those two processes are more separated between the cytoplasmic and the thylakoid membrane. This representation only serves the portrayal of the transcriptional changes and their putative affect on energy flow and not the actual distribution of the two chains between the thylakoid and the cytoplasmic membrane. Abbreviations: Temperature (Tm).

Photosynthetic electron transport and respiratory electron transport are alternative biochemical pathways that ultimately create a proton gradient that energizes F_0_F_1_ ATPase synthase. The thylakoid membranes of cyanobacteria are characterized by the simultaneous presence and intersection of the photosynthetic and respiratory electron transport chains, where several components are partially utilized in both processes [35]–[37]. Despite the spatial overlap of photosynthesis and respiration, the two electron chains offer alternative ways to adjust to environmental stresses. More precisely, this spatial overlap of the two electron transport chains in the thylakoids is more apparent in *Synechocystis*, while in *Synechococcus* the separation of the respiratory chain in the cytoplasmic membrane and the photosynthetic chain in the thylakoids is more defined [38]. Our data (Table S5) suggest that under certain conditions, photosynthetic and respiratory chain components were highly regulated (Figure 3A).

Transcriptional responses for the two electron transport chains under all stress conditions could be grouped into three major categories: 1) downregulation of photosynthetic electron transport chain genes and upregulation of the respiratory chain genes, 2) upregulation of the respiratory transport chain genes solely, 3) concurrent downregulation of genes from both electron transport chains, and 4) downregulation of the photosynthetic electron transport chain genes solely (Figure 3). The model in Figure 3 presents graphically how we speculate that these transcriptional alterations may impact the actual energy flow of the two strains in our selected conditions. Further experimental verification is required to establish this model.

### Upregulation of respiration and downregulation of photosynthetic related genes

Relative to reference conditions, stress conditions of low temperature, alkaline pH, and low light favored the upregulation of the respiratory-related genes in *Synechococcus* (Figure 3B, Table S5). For the last two conditions, this is summarized in the upregulation of certain gene components of the NDH-2 complex, NDH-1 dehydrogenase complex, and the two available terminal oxidases, the *αα_3_*-type cytochrome *c* oxidase (CoxBAC) and the cytochrome *bd* quinol oxidase (CydAB). In contrast, the transcript levels of the photosynthetic antenna and the photosystem I (PSI) related genes were reduced. In both stress conditions these alterations were evident both after 1-h and 24-h of exposure. Despite the fact that we found a similar pattern for low temperature in *Synechococcus* cells, this applies only for the first hour of exposure in the corresponding stress conditions, while after 24-h only respiration remained enhanced (see *Respiratory energy upregulation*).

The only example in *Synechocystis* where upregulation of the respiration-related genes prevailed over the photosynthetic ones is low temperature (24-h). More precisely, certain components of the NDH-1 dehydrogenase complex and the NDH-2 complex were induced, while the PSI and the antenna reduced.

### Upregulation of respiratory-related genes

*Synechococcus* cells after 24-h in low temperature exhibit an upregulation of certain components of the NDH-1 dehydrogenase complex and the NDH-2 dehydrogenase (see Table S5). This follows an enhanced respiratory and reduced photosynthetic transcriptional profile in the first hour of exposure. However, in carbon depletion we monitored elevated transcriptional levels of respiratory components only in the first hour of exposure, which is supported by an induction of the NDH-1 complex (but not the Nhd3 and nhF3 components), terminal oxidases, the cytochrome *b6f* complex and the F_0_F_1_-ATP synthase (Figure 3C).

### Simultaneous downregulation of photosynthetic and respiratory-related genes

*Synechocystis* cells 1-h after addition of 550 mM of NaCl exhibited a broad modification of expression levels for the majority of the genes that participate in the two electron transport chains. More precisely, 16 of the 32 components of the NDH-1 complex and all the three terminal oxidases (CydAB, CoxBAC and the quinol oxidase or QOX) were downregulated by 2- to 8-fold, while only 6 components were similarly upregulated. Interestingly, similar downregulation was also measured for almost all the components of the photosynthetic apparatus and certain components of the *b6f* cytochrome. Since both electron transport chains were downregulated, it is not surprising that the components of the F_0_F_1_ ATP synthase were also downregulated. However, transcript levels returned to normal 24-h after exposure to elevated NaCl levels, evidently showing an acclimation of *Synechocystis* cells to rapidly respond to elevated salinity. Our data are in agreement with previous microarray and proteomic data that subjected *Synechocystis* cells to salt stress for short and long periods [24], [28], [29], [30].

A similar, but less intense response was seen in *Synechocystis* for low light conditions. A 2- to 4.6-fold downregulation was seen for 43 to 47 components of the photosynthetic and respiratory electron transport chains for both time points, with only 2 genes upregulated (one of which degrades antenna protein). *Synechocystis* sp. PCC 6803 cells in low light seemed to be overall less affected compared to *Synechococcus* sp. PCC 7942, something that coincides with the photo-dependence differences these two strains exhibit, reported elsewhere [6], [7]. The fact that simultaneous downregulation of genes of the photosynthetic and respiratory electron transport chains was exclusively seen in *Synechocystis* sp. PCC 6803 cells may imply that this strain is either more adapted to survive with an impaired energy production and/or that employs other catabolic pathways to compensate for the energy loss (Figure 3D).

### Downregulation of photosynthetic-related genes

Ignoring upregulation of “respiratory” genes *ndhD3* and *ndhF3* (which actually participate in inorganic carbon transport) and the petA and petC1 genes (which are part of the shared cytochrome *b6f* complex) there was a general downregulation of “photosynthetic” genes in *Synechocystis* after 24 hours of exposure to Carbon limitation and 1-h to low temperature (Figure 3E), but never in *Synechococcus*. Photosynthesis provides the force for the essential carbon fixation pathway in photosynthetic organisms. The loss of the photosynthetic capacity as a result of the absence of inorganic carbon has been documented before in *Synechocystis* [31], [32]. Apparently, *Synechococcus* presents a different response to our tested conditions from *Synechocystis* at the indicated time points.

Overall, it is worth mentioning that *Synechocystis* cells often responded to stress with strong downregulation (up to 20-fold decrease) of F_0_F_1_-ATP synthase. This argues either for a lower energy requirement of the corresponding strain under stress conditions, or alternative pathways of energy acquisition.

### Carboxysome Operons

In *Synechocystis*, genes encoding CcmK1, CcmK2 and CcmL, which belong to the structural components that comprise the exterior of the capsule, are in the same positional cluster with the *ccmM* and *ccmN* genes that comprise the carboxysomal interior. Our data indicate that this cluster behaves as an operon since all components responded similarly to all tested conditions (see M&M regarding the definition of TUs). It is worth mentioning that the separate *ccmK3*/*ccmK4* cluster and the *ccmP* gene, encoding an outer shell facet protein, followed the trend of the CcmK1/K2/L/M/N cluster with the exception of 24-h in alkaline pH and 1-h of elevated salinity. Under these conditions, *ccmP* expression was unchanged while the other genes were all downregulated. The expression of the second outer shell facet CcmO component was mainly unaffected except for an upshift observed after 1-h in elevated salinity.

In *Synechococcus* the CcmK2/L/M/N cluster is adjacent to *ccmO* and two *rbcL,S* (RuBisCo) genes. However, the *ccmO* and the *rbc* genes seem to be transcripted independently from the CcmK2/L/M/N cluster. More precisely, the *rbc* genes exhibit a common pattern between them, placing them in their own operon, while *ccmO* is not coordinately regulated with either the CcmK2/L/M/N cluster or the rbc operon. Therefore, *ccmO* must form its own operon (Table S5). These observations are also seen in our 5′ data for *Synechococcus*.

Unlike in *Synechocystis*, we saw evidence in *Synechococcus* that it may alter carboxysome composition in response to different environmental conditions. After 1-h in low light, *ccmK3* and *ccmK4* in *Synechococcus* were downregulated while the CcmK2/L/M/N cluster was upregulated. Also, in most tested conditions *ccmK3* and *ccmK4* transcript levels in *Synechococcus* remained stable and did not follow the observed changes of the CcmK2/L/M/N cluster. Both of these *Synechococcus* responses in carboxysome structural protein gene expression were not seen in *Synechocystis*.

### Carboxysome gene expression

Overall, in *Synechocystis* the majority of the carboxysomal components were significantly repressed under several conditions, namely low light (1-h and 24-h), salinity stress (1-h), pH (24-h), and carbon limitation (24-h). On the other hand, in *Synechococcus* the *ccmK2/L/M/N* carboxysomal component had a trend to upregulation under alkaline pH (24-h) and low light (1-h), and slightly in elevated salinity (24-h) and carbon limitation (1-h and 24-h). Also, in *Synechococcus* the RuBisCo components also exhibited downregulation trends, under low temperature (1-h), alkaline pH (1-h) and low light (24-h) conditions, and were unchanged under all other conditions. These data indicate differences between the two strains in terms of protein and/or organelle stability under the tested conditions.

The NdhD3/F3 complex and the SbtA transporter were the most upregulated inorganic carbon transport systems for both strains, although the specific conditions causing upregulation varied. In *Synechocystis* upregulation occurred under low temperature (1-h), alkaline pH (1-h) and 24-h after carbon limitation. In *Synechococcus* upregulation occurred under low light (1-h), low temperature (24-h) and alkaline pH (1-h and 24-h) conditions. The fact that no induction of the major inorganic carbon transport systems was observed after carbon limitation in *Synechococcus*, could be due to the fact that either the carbon response is not yet fully activated in the tested time window (1-h and 24-h), or that they responded sometime between the 1-h to 24-h [39].

## Conclusions

In this report we have employed an Illumina RNA-seq study for an in depth analysis of the transcriptome of the two cyanobacteria *Synechococcus* and *Synechocystis*. Using this data we were able to verify and improve the structural annotation of both genomes by detecting new transcripts located in the intergenic regions. Additionally gene annotations in *Synechoccocus* were improved based on 5′ enriched libraries evidence, and transcriptional units (operons) were identified containing two genes on average. The identification of genes with similar expression profiles under the conditions examined allowed us to link genes with undetermined function to annotated ones and their corresponding pathways.

Overall, comparative analysis of the response of the two organisms revealed similarities but also significant changes in the way they acclimate to external stress at two specified time points representing a short (1-h) and long term acclimation (24-h). In *Synechococcus* the majority of the stress conditions resulted in the upregulation of the respiratory genes and the downregulation of the photosynthetic ones. This indicates that the loss of energy production that results from a sensitive photosynthetic electron transport chain is compensated by the induction of the respiratory one and suggests a more robust interaction between them in this organism. On the other hand, in *Synechocystis* there were cases that genes of both electron transport chains were downregulated. The carboxysome, a crucial organelle for carbon fixation, exhibits differences between the two strains. *Synechococcus* cells likely enhance the carboxysomal capsule in a number of environmental stresses, while *Synechocystis* does not.

We believe that this work will provide a valuable reference for further studies of cyanobacterial transcription and acclimation to environmental stress.

## Supporting Information

Table S1 **Read mapping statistics for the different samples.**
(XLSX)

Table S2 **RPKM levels for genes of both organisms for all conditions.** Scale: RPKM*10∧9.
(XLSX)

Table S3 **Results obtained by using 5′ enriched data for** ***Synechococcus*** **.** Individual worksheets list CDS with 5′ peak downstream of their annotated translation initiation sequence, CDSs without any detectable 5′ peak and Transcriptional Units with more than one CDS (operons).
(XLS)

Table S4 **CDS that could be functionally related using unsupervised clustering based on the assumption that genes with similar expression profiles are functionally linked.**
(XLSX)

Table S5 **Subset of Supplementary Table S2.** Gene expression data for genes associated with pathways referenced in the manuscript.
(XLSX)

File S1 **Sequence similarity results based on blast for newly identified transcribed sequences located in intergenic regions.**
(ZIP)
